# Atypical Cystic Primary Hepatic GIST: A Case Report of Rare Presentation and Long-Term Survival

**DOI:** 10.3390/curroncol32070383

**Published:** 2025-07-01

**Authors:** Mirela Claudia Rimbu, Florin Dan Ungureanu, Cosmin Moldovan, Madalina Elena Toba, Marinela Chirila, Elena Truta, Daniel Cord

**Affiliations:** 1 Medical Doctoral School, Titu Maiorescu University of Bucharest, 040317 Bucharest, Romania; mirela.rimbu@prof.utm.ro (M.C.R.); florin.ungureanu@prof.utm.ro (F.D.U.); daniel.cord@prof.utm.ro (D.C.); 2 Department of General Surgery, Witting Clinical Hospital, 010243 Bucharest, Romania; cosmin.moldovan@prof.utm.ro (C.M.); madalina.toba@prof.utm.ro (M.E.T.); 3 Faculty of Pharmacy, Titu Maiorescu University, Gheorghe Sincai Blv. 16, 040314 Bucharest, Romania; 4 Departament of Pharmacy, Emergency Clinical Hospital Bucharest, 8. Floreasca Street, 014461 Bucharest, Romania

**Keywords:** primary hepatic gastrointestinal stromal tumours, tyrosine kinase inhibitors, imatinib, sunitinib

## Abstract

Primary hepatic gastrointestinal stromal tumours (PHGISTs) are rare and frequently misdiagnosed due to their atypical presentation and uncertain origin. The purpose of this article is to present the case of a 79-year-old female patient with a gigantic PHGIST characterized by a predominantly cystic nature—an extremely rare presentation, as most cases of PHGIST are solid. Despite extensive imaging and exploratory laparotomy, the primary origin remained uncertain, leading to questioning about whether it was a true primary hepatic GIST or an atypical metastatic lesion. The initial therapeutic approach involved a surgical procedure aimed to confirm the diagnosis and achieve reductive tumourectomy. Following the surgery, the patient was administered imatinib with a favourable clinical response for four and a half years—an atypical pattern of resistance, as most patients typically develop therapeutic resistance within two to three years. A second surgical intervention was performed to address a cystic lesion localized in the left hepatic lobe, followed by an atypical segment III hepatectomy to achieve macroscopic resection. Subsequently, the patient received sunitinib for two and a half years, which resulted in temporary disease stabilization. However, the sunitinib treatment was associated with hypertension and leukopenia. The patient’s overall survival was 8 years, suggesting that individualized therapeutic strategies and close monitoring might be the key in such cases. Furthermore, this case confirms the role of surgical intervention even in advanced disease stages, with multiple major resections contributing significantly to prolonged survival. The interplay between surgical and oncologic therapies remains essential to guiding clinical decisions. Given the unusual cystic presentation, this case highlights the necessity to expand the pathological and molecular profiling of PHGISTs. Furthermore, the atypical timeline of resistance development and treatment-related toxicity emphasizes the importance of further research into the genetic and pharmacological determinants of PHGISTs. These findings advocate for the refinement of diagnostic, therapeutic, and surveillance protocols tailored to rare GIST subtypes.

## Simple Summary

Primary hepatic gastrointestinal stromal tumours (PHGISTs) are rare and often misdiagnosed because of their unusual liver location. Typically, these tumours are solid and have a poor prognosis. This article presents a unique case of a 79-year-old woman with a mostly cystic PHGIST who survived for eight years, challenging the usual understanding of these tumours. Her long survival was due to personalized treatment, including multiple surgeries and targeted therapies, showing that individualized care leads to better outcomes. These findings suggest that PHGISTs can appear in unexpected forms, indicating the need for a team approach and continuous monitoring. Additionally, the patient developed resistance to initial treatments, emphasizing the importance of ongoing research into genetic and molecular characteristics to develop more effective therapies. Future research should focus on developing advanced imaging techniques and genetic testing to improve early detection and treatment. Integrating personalized treatment plans and multidisciplinary care could significantly enhance patient outcomes.

## 1. Introduction

Gastrointestinal stromal tumours are the most common form of mesenchymal tumours of the digestive tract, originating in the interstitial Cajal cells. They usually occur in extrahepatic locations, but a small percentage originate in the liver and are referred to as primary hepatic gastrointestinal stromal tumours (PHGISTs) [1,2]. Vague symptoms often result in a late diagnosis and poor prognosis, with about 20% of patients presenting metastases at diagnosis. This underscores the urgent need for increased awareness among healthcare professionals regarding this disease [2].

PHGISTs are increasingly recognized as a challenging subset of gastrointestinal stromal tumours due to their atypical presentation, their poor prognosis, and the difficulties associated with establishing an accurate diagnosis [3]. Generally, these tumours are discovered by chance, occur sporadically, and are associated with KIT and PDGFRA gene mutations [4].

The diagnosis of PHGISTs is made by exclusion, as they present the same molecular immunohistochemical and histological features as classical gastrointestinal stromal tumours. Imaging techniques such as PET-CT are necessary to exclude the presence of metastases from GISTs before the diagnosis of a PHGIST is established. Multiparametric MRI and PET scans have improved the ability to differentiate PHGISTs from other hepatic lesions, ultimately aiding in treatment planning [5]. Although imaging techniques such as PET-CT are preferred for comprehensive evaluations, the initial diagnostic work-up for the patient in this case study consisted of abdominal ultrasound, standard endoscopies, and CT scans, which were deemed sufficient for the clinical context.

Tyrosine kinase inhibitors (KTI) are the standard therapy being used either in combination with surgery or as a monotherapy [6,7]. Immunohistopathology may contribute to the development of screening recommendations and assessment of treatment resistance. The combination of surgery and tyrosine kinase inhibitor therapy has been proved to be safe and to improve outcomes, particularly in the adjuvant setting [8].

Other therapeutic strategies include transarterial chemotherapy and tumour ablation, which are considered palliative options. Furthermore, recent studies showed promising results for plant extracts with silver nanoparticles that might have the potential to minimize the side effects and, at the same time, increase the effectiveness of tyrosine kinase [9].

Due to the small number of published studies, information on the morbidity and mortality associated with PHGISTs is still limited [1].

## 2. Case Report

A 71.5-year-old female patient (at the time of diagnosis) with a history of heavy smoking (one pack per day for 55 years) and family history of oncological diseases presented with a loss of appetite, pronounced fatigue, moderate weight loss, moderate diffuse abdominal pain, dyspnoea, and a moderate cough. The symptoms had an insidious onset, lasting for six months before the patient sought medical attention. A medical examination revealed an enlarged abdomen (symmetrical distension, a large amount of tense ascites, and intact intestinal transit for gas and faeces, without changes in appearance or consistency) and restrictive respiratory dysfunction.

### 2.1. Timeline Presentation

Month 0 Diagnosis of primary hepatic gastrointestinal stromal tumour (PHGIST).
Month 1 First surgical intervention to confirm diagnosis and perform reductive tumourectomy.
Month 3 Initiation of first-line treatment with imatinib (400 mg/day).
Month 64 Second surgical intervention for cystic tumour in the left hepatic lobe, followed by third surgical intervention involving atypical segment III hepatectomy.
Month 65 Initiation of second-line treatment with sunitinib (50 mg/day).
Month 72 CT evaluation shows stable hepatic disease; side effects noted.
Month 85 New cystic lesion detected in segment VII, raising concerns about treatment resistance.
Month 96 Patient’s death recorded, 8 years post-diagnosis.

### 2.2. Initial Diagnosis

The initial biochemical evaluation showed moderate hypochromic microcytic anaemia, thrombocytosis, mild hepatic cytolysis, anicteric cholestasis syndrome, and a significant inflammatory syndrome. Biochemical and microbiological tests excluded infection with HBV or HCV, intestinal parasitosis, or occult gastrointestinal bleeding.

A chest X-ray showed significant elevation of the right hemidiaphragm without other notable changes. An abdominal–pelvic ultrasound indicated a heterogeneous liver texture with multiple anechoic lesions, including two large ones: 9 × 13.7 cm in the left hepatic lobe and 7 × 11 cm in the right hepatic lobe.

**Figure 1:**
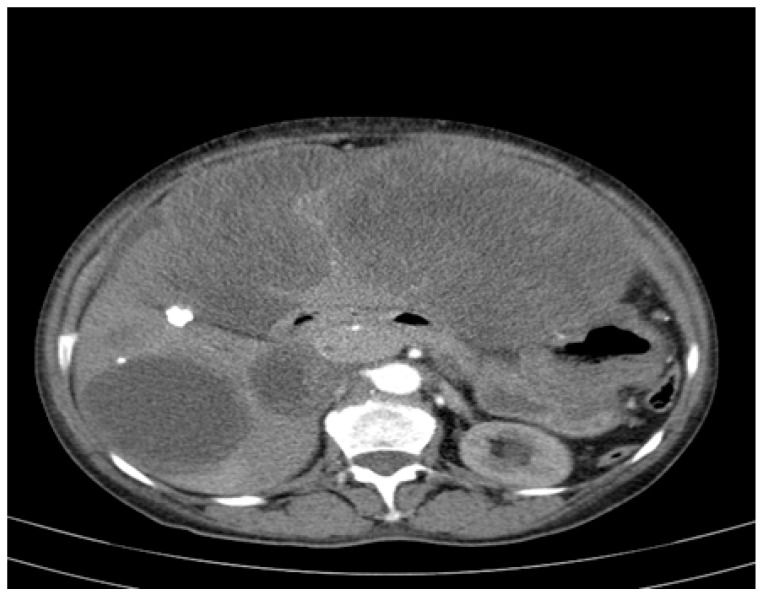
CT appearance at the time of diagnosis: hepatic tumour formations with a cystic morphology, showing walls of variable thickness and heterogeneous contrast enhancement.

An abdominopelvic CT revealed liver enlargement due to the presence of numerous cystic lesions ranging in size from 1 cm to 18 cm, most of them located in the right hepatic lobe (Figure 1). Two of the largest cystic formations were located at the inferior liver margin, one in segment IVb and the other in segment III of the left hepatic lobe, occupying almost the entire abdominal cavity. The inferior pole of the cystic formation arising from the left hepatic lobe extended into the pelvic cavity (14 × 18 × 18.5 mm AP/Transverse/CC), displacing the transverse colon posteriorly and shifting the jejuno-ileal loops toward the right iliac fossa. The cysts had well-defined contours, but their inner walls varied in thickness and exhibited heterogeneous contrast enhancement. Three hepatic parenchymal calcifications were present in the right hepatic lobe (segments V and VI), with the largest measuring 14 mm and the smallest 2–3 mm; two of them were adjacent to the cystic wall. A thin layer of perihepatic/hepatodiaphragmatic fluid was also noted, with an uneven thickness ranging from 1 to 2.3 cm.

Upper and lower gastrointestinal endoscopy were performed, revealing no pathological changes but with an inability to evaluate the small intestine. Hepatic hydatidosis was suspected due to a positive IgG test for *Echinococcus granulosus*.

The patient was admitted to the General Surgery Department of CF1 Witting Hospital in Bucharest, where a surgical intervention was planned to confirm the diagnosis and to perform a reductive tumourectomy.

### 2.3. First Surgical Intervention

**Figure 2:**
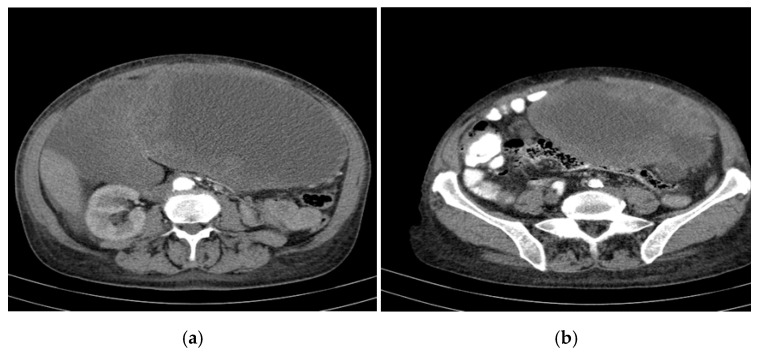
CT appearance at the time of diagnosis: the inferior pole of the cystic formation in the left hepatic lobe extended into the pelvis (14/18/18.5 cm), displacing the transverse colon and small intestine posteriorly toward the right iliac fossa. (**a**) upper abdomen section on the left (**b**) pelvic section on the right.

Preintervention, a re-evaluation of the intrahepatic cystic formations (correlated with the CT findings) was performed and revealed the following:

- Segment VII: Two target-like (bull’s-eye) images with a hypoechoic/transonic peripheral rim and a heterogeneous hyperechoic central zone, with maximum diameters of 3.1 cm and 1.8 cm.
- Portal vein: Measured 8 mm; the common bile duct (CBD) was 5 mm with heterogeneous content.
- Gallbladder: Compressed gallbladder, non-identifiable.
- Peripheral hepatic parenchyma: Exhibited steatosis.
- Large cystic formations: Occupying the right hypochondrium and flank, epigastrium, left hypochondrium, and left flank, with a transonic appearance, thickened heterogeneous wall (up to 3 cm), and irregular hyperechoic septations, some possibly vascularized.
- Largest transonic formation: Located in the left abdomen with a heterogeneous circumferential wall (up to 8.3 in thickness); contained heterogeneous fluid with multiple echogenic/hyperechoic echoes, which were mobile upon compression (Figure 2).

An exploratory laparotomy was performed through a midline xiphopubic incision, revealing approximately 2 L of clear ascitic fluid. Hepatic tumour formations had nearly replaced both liver lobes, creating large cystic cavities. The cystic tumour in the left lobe extended to the left iliac fossa, appeared friable, bled profusely, and was adherent to the intestinal mass, rendering it immobile. The remaining liver parenchyma was only palpable at the periphery of segments VI–VII, with evidence of tumour infiltration in the round ligament.

An excisional biopsy collected 1–2 cm tissue fragments from the right hepatic lobe tumour, and the left cystic tumour was incised and evacuated, releasing about 500 mL of clear fluid. Tumour debulking was performed, but significant haemorrhaging occurred, which was managed using compression and haemostatic sponges.

The postoperative course was initially slow, with significant haemorrhagic ascitic fluid losses through the peritoneal drains, necessitating repeated transfusions of packed red blood cells. The patient was discharged 30 days after admission, presenting with a healed surgical site, active intestinal transit, and oral intake, although the peritoneal drain persisted, with an output of approximately 500 mL/day of ascitic fluid.

### 2.4. Histopathological Examination

The histopathological evaluation revealed tumour tissue fragments composed of spindle cells with clear vacuolated cytoplasm and relatively homogeneous oval nuclei, displaying hyperchromatic and vacuolated features, with nucleoli that were difficult to identify. The cellular boundaries were indistinct, and the cells were arranged around ectatic thin-walled blood vessels. The mitotic activity was low (3 mitoses/50 HPF). The histopathological analysis concluded that the tumour was a clear cell proliferation of mesenteric origin, suggestive of a gastrointestinal stromal tumour (GIST).

### 2.5. Immunohistochemical Analysis

To confirm the diagnosis, tumour fragments were analysed using a broad panel of immunomarkers, and the results were as follows:

- CD117, VIMENTIN, PDGFR-Alpha, CD34, DOG1—Positive in tumour cells.
- ACTININ—Negative in tumour cells, positive in blood vessels.
- Ki67—Positive, with a proliferation index of 25% in tumour cells.
- S100—Negative.

The immunohistochemical diagnosis was malignant gastrointestinal stromal tumour (GIST) of the liver.

### 2.6. First-Line Treatment with Imatinib (Total Duration: 55 Months)

Treatment with imatinib (GLIVEC 100 mg tablets) was initiated as a 400 mg/day, single-dose administration in the morning. Treatment initiation led to a rapid improvement in the patient’s general condition, a reduction in drainage to 50 mL/24 h, and a decrease in abdominal volume; drainage tubes were removed approximately one month after the initiation of therapy.

A CT evaluation at discharge revealed multiple secondary cystic hepatic lesions, the largest located in the left lobe; a contrast enhancement anomaly in segment VII; a small amount of ascitic fluid; and postoperative changes in the anterior abdominal wall, in contact with the cystic formation in segments II–III.

**Figure 3:**
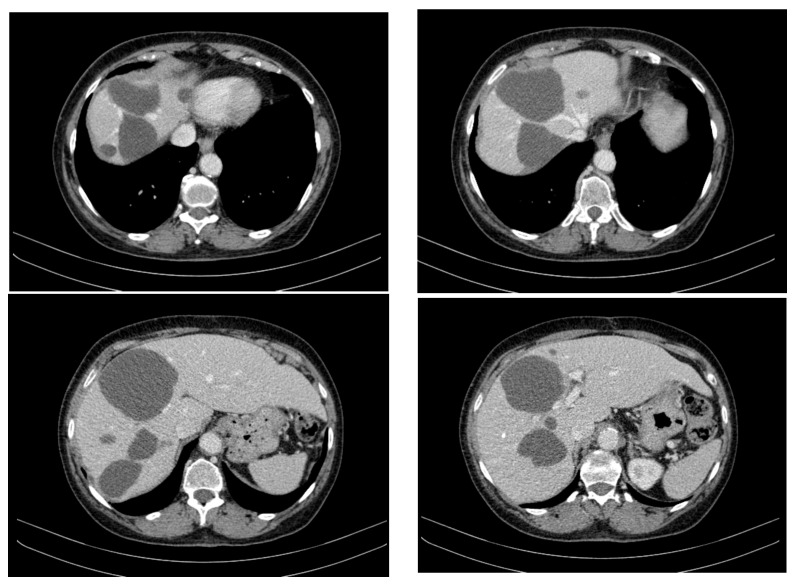
CT appearance at 12 months of treatment with imatinib at different abdominal levels.

Over the course of 55 months of treatment with imatinib, the patient was monitored by imaging, exhibiting a stable disease course with no significant tumour progression and some evidence of partial regression of specific lesions. CT appearance at 12 months of treatment with imatinib is presented in Figure 3. The following key observations emerged from the serial CT evaluations:

- Hepatic tumour stability: Multiple large cystic hepatic lesions remained stable in size throughout the monitoring period, with no significant new secondary lesions developing. The largest hepatic cystic tumours persisted in segments IV, VI, and VIII, with maximum dimensions of ~8 cm, without major structural changes.
- Regression of extrahepatic lesions: Initial gastric and peritoneal lesions, including a cystic formation in the anterior gastric wall and ascitic fluid, regressed significantly by month 14 and did not reappear in later scans. The left inguinal hernia, although noted consistently, remained unchanged, without complications.
- Emergence of new findings without aggressive progression: A subcapsular nodule (3.5 × 2 cm) in Morrison’s space was identified during month 41, remaining stable without concerning enhancement patterns. A pseudo-nodular perfusion anomaly in segment VIII was noted but was attributed to pressure from the large cystic tumour rather than neoplastic progression. Mild gastric antral wall thickening developed later (by month 49), with a maximum thickness of 14 mm, but remained stable thereafter, with no associated adenopathy or signs of malignant transformation.
- Absence of significant distant metastases or new systemic involvement: Throughout the entire monitoring period, there were no newly detected secondary metastatic lesions in the thoracic, abdominal, or pelvic regions; no significant adenopathy was identified; and there was no recurrence of ascitic fluid beyond a mild amount in the early monitoring phases.

The patient received the following concomitant medication: ferrous sulphate (80 mg, 1 tablet/day) for anaemia; esomeprazole (20 mg, 1 capsule/day) to reduce gastric acid production; a fixed drug combination of tramadol and paracetamol (37.5 mg/325 mg), as needed, up to 3 times/day for pain management; and LIV. 52 (4 times/day), a herbal supplement used to support liver function.

The patient experienced intense muscle spasms in the lower limb muscles, particularly in the triceps surae. The frequency of these spasms progressively increased from once per month in the early months of imatinib treatment to three times per week in the later stages of treatment.

The patient also began to display sporadic, low-volume vomiting.

The CT scan at 63 months post-diagnosis revealed a newly developed large cystic tumour, in contrast to the previously stable disease course. The key findings compared to the CT at 56 months were

- New large cystic tumour formation;
- Previously known lesions remaining stable;
- An absence of metastatic progression.

The appearance of a new, large cystic tumour, particularly with a thicker wall and connection to both the liver and gastric antrum, raised concerns of tumour evolution despite the prior stability. The absence of new distant metastases and the stability of previously known lesions indicated that the disease remained relatively controlled overall, but this new growth warranted immediate further evaluation.

The tyrosine kinase inhibitor (TKI) treatment was discontinued, and the patient was surgically re-evaluated at CF1 Witting Hospital, Bucharest; the patient did not report significant subjective complaints, other than sporadic, low-volume vomiting and muscle spasms. Laboratory tests, chest X-ray, upper gastrointestinal endoscopy, and a cardiology consultation were performed, revealing no significant pathological changes, except for moderate iron deficiency anaemia. The biochemistry results revealed chronic normochromic, normocytic anaemia (Hb = 8.4 g/dL).

### 2.7. Second Surgical Intervention

The second surgery was performed for the cystic tumour in the left hepatic lobe, which measured approximately 12 × 15 cm and exhibited a tense, fibrous wall. An incision was made to evacuate about 1000 mL of old blood, followed by intraoperative lavage and drainage.

Postoperatively, the patient experienced persistent venous bleeding through the drainage tubes (500–750 mL/24 h), necessitating multiple blood transfusions and fluid resuscitation. Despite these efforts, significant blood loss continued, with an output of 300–700 mL/24 h through the drains, and the patient’s haemoglobin dropped to 9 g/dL.

### 2.8. Third Surgical Intervention

The patient was transferred to another hospital, Fundeni Clinical Institute (ICF), to its General Surgery Clinic for further management and underwent a third surgical reintervention to remove a large cystic tumour in segment III. An atypical segment III hepatectomy was performed, removing the tumour en bloc.

Postoperatively, the patient’s condition improved. She remained afebrile, normal intestinal transit resumed, and the drainage output progressively decreased, allowing for the removal of the peritoneal drainage tubes after a follow-up ultrasound. The ultrasound revealed remaining liver with a heterogeneous echotexture due to macronodular lesions but no signs of biliary obstruction or ascites. The patient was discharged in good general condition, with a healing surgical wound.

### 2.9. Second-Line Treatment with Sunitinib (Total Duration: 30 Months)

The histopathological examination from the second surgery confirmed a diagnosis of a high-grade malignant GIST. At approximately 5.5 years post-diagnosis, the patient began second-line therapy with sunitinib (50 mg/day for 28 days followed by a 14-day break).

**Figure 4:**
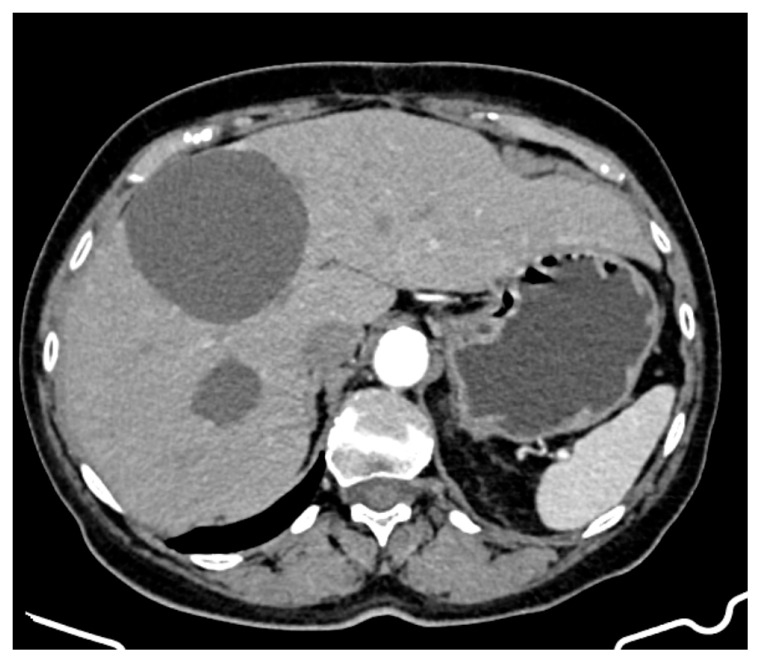
CT appearance at 7 months of treatment with sunitinib.

A CT evaluation after 7 months of sunitinib therapy showed stable hepatic disease, with no new metastatic lesions (Figure 4). However, the patient experienced significant side effects, including Grade 3 neutropenia and hypertension, leading to temporary treatment interruptions. Other reported side effects included cataract development, complete nail bed dekeratinization of the toes, and persistent anaemia, for which she received supportive therapy.

**Figure 5:**
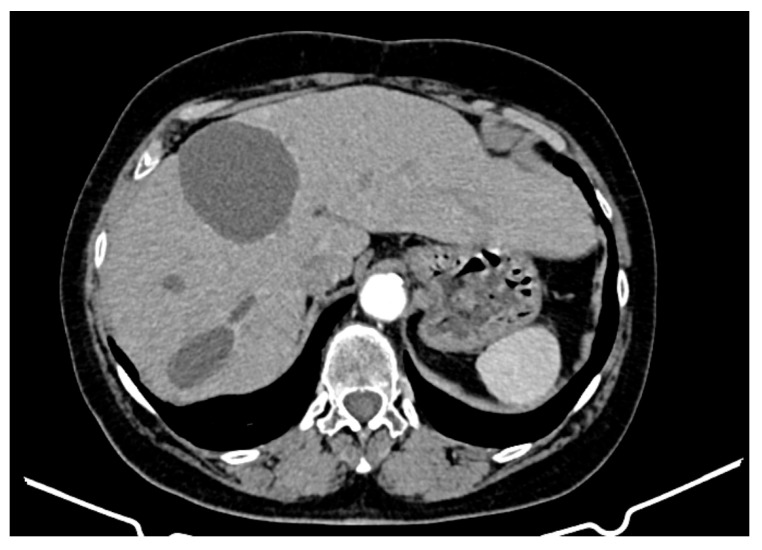
CT appearance at 20 months of treatment with sunitinib.

At 20 months on sunitinib (85 months post-diagnosis), a new cystic lesion in segment VII was detected, raising concerns about treatment resistance (Figure 5). While some known lesions had slightly decreased in size, the emergence of this new lesion indicated ongoing tumour activity that required close monitoring and possible re-evaluation of the treatment strategy.

### 2.10. Summary of Clinical–Pathological Data

Initial Presentation
  Age: At diagnosis, 71.5 years.
  Symptoms: Loss of appetite, fatigue, weight loss, abdominal pain, dyspnoea, cough.
  Biochemical Evaluation: Moderate anaemia, thrombocytosis, mild hepatic cytolysis, anicteric cholestasis syndrome, significant inflammatory syndrome.
  Imaging: Abdominopelvic CT showed numerous cystic liver lesions (1 cm to 18 cm), mostly in the right hepatic lobe. Largest cysts in segments IVb and III of the left hepatic lobe.
First Surgical Intervention
  Findings: Large cystic formations with a transonic appearance and thickened heterogeneous walls. Portal vein measured 8 mm; CBD 5 mm with heterogeneous content.
  Histopathology: Spindle cells with vacuolated cytoplasm and oval nuclei. Low mitotic activity (3 mitoses/50 HPF).
  Immunohistochemistry: Positive for CD117, VIMENTIN, PDGFR-Alpha, CD34, and DOG1; Ki67 proliferation index of 25%.
Treatment with Imatinib (55 months)
  Dosage: 400 mg/day.
  Response: Rapid improvement, reduced drainage, stable disease course with partial regression of lesions.
  Side Effects: Muscle spasms, sporadic vomiting.
Second Surgical Intervention
  Procedure: Addressed cystic tumour in the left hepatic lobe.
Third Surgical Intervention
  Procedure: Atypical segment III hepatectomy for a large cystic tumour.
Treatment with Sunitinib (30 months)
  Side Effects: Hypertension, neutropenia, nail bed dekeratinization in all toes, alopecia, persistent anaemia.
Disease Progression and Outcome
  Survival: Eight years post-diagnosis.
  Final Symptoms: Abdominal distension, pain, paraesthesia in limbs.

### 2.11. Disease Progression and Outcome

The patient’s death was recorded at home 96 months after the initial diagnosis (corresponding to 8 years of disease progression). No post-mortem examination was performed to determine the precise cause of death.

In the months before passing, the patient reported worsening symptoms, including abdominal distension with increased volume, diffuse abdominal pain, lower back pain, severe bloating, diffuse limb pain with paraesthesia in the lower limbs, and pain refractory to previously prescribed analgesics, suggesting escalating discomfort and potential treatment resistance.

The progressive worsening of these symptoms, particularly uncontrolled pain, abdominal distension, and neurological symptoms (paraesthesia, limb pain), suggested a significant tumour burden and possible complications such as ascites, nerve compression, or systemic disease progression.

The exact cause of death remained undetermined, but potential factors could have included hepatic or multiorgan failure, progressive tumour-related complications, or systemic effects of long-term disease progression.

This marked the end of an 8-year disease course, during which the patient underwent multiple lines of treatment, surgical interventions, and disease monitoring.

## 3. Discussion

Primary hepatic gastrointestinal stromal tumours (PHGISTs) are rare neoplasms with atypical clinical presentations, often leading to diagnostic challenges and delayed treatment initiation. They often present atypically and may be mistaken for metastatic disease rather than a primary hepatic tumour. Immunohistochemistry and molecular analysis are essential for accurate diagnosis.

This case illustrates a rare presentation of PHGIST characterized by predominantly cystic features and a long disease course, including resistance to imatinib and significant side effects from TKI therapies. Given the rarity of predominantly cystic PHGISTs, this case underscores the necessity for individualized diagnostic and treatment approaches [5].

Despite the absence of a clear primary site in the gastrointestinal tract, the immunohistochemical findings suggested a hepatic origin, though some researchers argue that such tumours may represent metastatic extensions from gastrointestinal GISTs rather than true hepatic primaries. In such cases, comprehensive endoscopic and imaging evaluations during diagnostic work-up are very important. For the present case, abdominal ultrasound, standard endoscopies, and CT scans were considered sufficient. In addition, immunohistochemistry and molecular profiling that includes an analysis of mutations in KIT and PDGFRA can aid in differentiating between primary hepatic tumours and metastases.

Preoperative tissue sampling under percutaneous or endoscopic ultrasound guidance is crucial for accurately diagnosing atypical tumours like primary hepatic gastrointestinal stromal tumours (PHGISTs). This method enhances diagnostic precision and aids in distinguishing between primary and metastatic lesions, which is vital for effective treatment planning. As noted in the literature, ultrasound-guided sampling significantly improves diagnostic outcomes [10].

The patient exhibited prolonged survival of eight years, significantly exceeding the median survival reported in the literature (a five-year survival rate of approximately 33%) [4]. The factors that contributed to this long survival period included regular imaging monitoring, individualized treatment adjustments, and successful surgical interventions.

Initially stable, the cystic liver tumours progressed over time, necessitating major surgical resections, including an atypical hepatectomy for a large 12 cm cystic lesion. The patient responded well to imatinib for four and a half years before developing resistance, a phenomenon typically reported after two to three years [11,12]. Severe muscle spasms were the primary adverse effect associated with imatinib. Following imatinib resistance, treatment with sunitinib was initiated and maintained for two years until disease progression.

A study by Mohammadi M et al. (2025) indicated that different mutational profiles significantly influence treatment responses and surgical outcomes in GIST patients receiving neoadjuvant imatinib. Therefore, mutational profiling might be a useful tool in guiding treatment decisions and optimizing outcomes for GIST patients undergoing neoadjuvant therapy [13].

Resistance to first-line TKIs is a known challenge in GIST treatment and, thus, alternative treatments are under investigation. mTOR inhibitors (such as everolimus or temsirolimus) are studied for their potential to overcome resistance in cases of GISTs. A combination of TKIs and mTOR might provide increased efficacy and delay resistance onset [14]. Current research efforts focus on creating new drugs that target the entire range of KIT mutations to enhance the inhibition of this oncogenic pathway [15].

Although the patient demonstrated initial stability, significant toxicity was observed, including moderate to severe hypertension, nail bed dekeratinization, alopecia, binocular cataract development, transient leukopenia, and progressive arterial stenosis. The adverse effects experienced with sunitinib are less commonly reported in similar cases but align with the known vascular and cutaneous toxicities of TKIs [16].

Severe side effects from TKIs may impact treatment adherence. Muscle spasms (caused by imatinib), hypertension, cataracts, and vascular complications (caused by sunitinib) significantly affected the patient’s quality of life, underlining the importance of proactive side effect management.

Surgery remains a critical treatment option for GISTs, especially if compared to other rare and aggressive cancers like mesothelioma (which shares a common PI3K/AKT/mTOR activation pathway) where surgery has no or a limited impact on survival rates, even if applied in the early stages of the disease [17]. In contrast, surgical resection of large GISTs, when feasible, can improve survival and potentially delay progression even in advanced stages of the disease. As reported by Ryu and Kim (2020), liver resection significantly improves survival rates, suggesting that surgical management remains a critical component of treatment even in the context of advanced disease and previous therapeutic failures [18].

This case underscores the challenges in PHGIST management and highlights the importance of a multidisciplinary approach combining surgery and systemic therapy. This is in line with the results of a study by Ran P. et al. (2023), which demonstrated that multidisciplinary discussions are a valuable strategy for enhancing the outcomes of patients with advanced gastrointestinal stromal tumours (GISTs) [19].

The patient’s prolonged survival despite an aggressive disease course suggests that individualized therapeutic strategies, continuous monitoring, and timely surgical interventions can enhance outcomes. Recent studies also showed that monitoring patient responses to therapy and using personalized treatment approaches, tailored based on individual genetic profiles and tumour characteristics, are important to enhancing the effectiveness of GIST management [20].

Further research is needed to refine diagnostic criteria, optimize treatment sequencing, and identify markers of resistance to improve prognostic predictions and therapeutic efficacy in PHGIST management.

## 4. Conclusions

PHGISTs represent a complex medical challenge that requires a multidisciplinary approach. This case report described a patient with a predominantly cystic tumour and an atypical clinical course characterized by late resistance to imatinib and prolonged survival. Imatinib was used successfully for four years until resistance developed. Sunitinib provided a temporary stabilization of the disease, but it was associated with serious adverse drug reactions. The personalized and multidisciplinary approach (involving surgery, oncological treatment, and close monitoring) played an important role in the prolonged patient survival. This case demonstrates that there is potential for prolonged survival even in rare diseases such as PHGISTs but also highlights the severe side effects associated with standard therapies that limit their use.

## Data Availability

The raw data supporting the conclusions of this article will be made available by the authors on request.

## Abbreviations

The following abbreviations are used in this manuscript:

CBD	Common bile duct
CT	Computer tomograph
GIST	Gastrointestinal stromal tumour
IHC	Immunohistochemistry
PHGIST	Primary hepatic gastrointestinal stromal tumour
TKIs	Tyrosine kinase inhibitors
